# Microtubule-Actin Crosslinking Factor 1 and Plakins as Therapeutic Drug Targets

**DOI:** 10.3390/ijms19020368

**Published:** 2018-01-26

**Authors:** Quincy A. Quick

**Affiliations:** Department of Biological Sciences, Tennessee State University, 3500 John A. Merritt Blvd, Nashville, TN 37209, USA; qquick@tnstate.edu; Tel.: +1-(615) 963-5768

**Keywords:** plakins, drug targets, cancer, cardiomyopathy, bullous pemhigoid

## Abstract

Plakins are a family of seven cytoskeletal cross-linker proteins (microtubule-actin crosslinking factor 1 (MACF), bullous pemphigoid antigen (BPAG1) desmoplakin, envoplakin, periplakin, plectin, epiplakin) that network the three major filaments that comprise the cytoskeleton. Plakins have been found to be involved in disorders and diseases of the skin, heart, nervous system, and cancer that are attributed to autoimmune responses and genetic alterations of these macromolecules. Despite their role and involvement across a spectrum of several diseases, there are no current drugs or pharmacological agents that specifically target the members of this protein family. On the contrary, microtubules have traditionally been targeted by microtubule inhibiting agents, used for the treatment of diseases such as cancer, in spite of the deleterious toxicities associated with their clinical utility. The Research Collaboratory for Structural Bioinformatics (RCSB) was used here to identify therapeutic drugs targeting the plakin proteins, particularly the spectraplakins MACF1 and BPAG1, which contain microtubule-binding domains. RCSB analysis revealed that plakin proteins had 329 ligands, of which more than 50% were MACF1 and BPAG1 ligands and 10 were documented, clinically or experimentally, to have several therapeutic applications as anticancer, anti-inflammatory, and antibiotic agents.

## 1. Introduction

**Figure 1:**
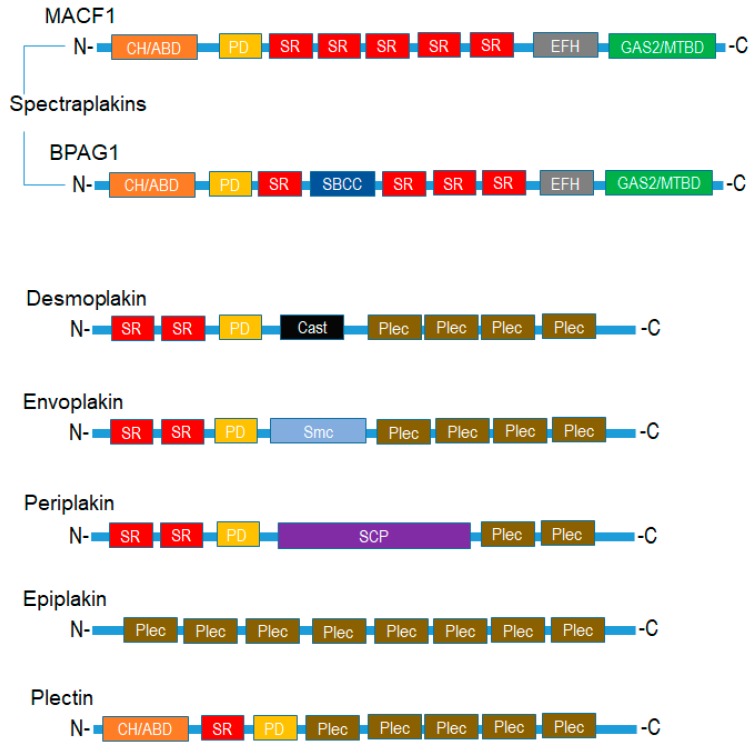
Domain regions and organizational structure of plakin proteins. CH-ABD: calponin homology actin-binding domain; SR: spectrin repeats; EFH: EF-hand calcium-binding domain; GAS2-MTBD—growth-arrest specific protein 2 microtubule binding domain; Plec: plectin; SBCC: DNA repair exonuclease SbcCD ATPase subunit domain; PD: Plectin domain; Cast: RIM-binding protein of the cytomatrix active zone; Smc: Chromosome segregation ATPase; SCP: Synaptonemal complex protein 1.

Mammalian plakins are a family of seven cytoskeletal proteins (microtubule-actin crosslinking factor 1-MACF1, bullous pemphigoid antigen 1-BPAG1, plectin, desmoplakin, envoplakin, periplakin, and epiplakin) that were collectively identified and discovered between the early 80s and mid-90s [1,2,3,4,5,6,7]. Despite their discovery over thirty years ago, much still remains to be determined regarding the biological function of plakins. It is well documented that plakins and their isoforms, which arise via alternative splicing mechanisms and different promoter usage, interact with and serve as cross-linkers of microtubules, intermediate filaments, and microfilaments. Further complexity regarding plakin protein isoforms, as it relates to the structural and functional diversity of this complex protein family, can be observed with the varying number of isoforms identified. Plectin has the greatest number of isoforms, i.e., 12, desmoplakin 2, MACF1 6, and BPAG1 3 major isoforms. MACF1 and BPAG1 perform crosslinking functions between actin and microtubules, while plectin crosslinks intermediate filaments to microtubules, and desmoplakin, envoplakin, periplakin, and epiplakin are predominately involved in intermediate filament binding as components of desmosomes and the cornified envelope. Mammalian plakins are comprised of several varied structural and binding domains that consist of a combination of actin-binding domains, plakin domains, spectrin repeats, coiled-coil rods, EF-hand calcium-binding domains, and growth-arrest specific 2-related microtubule-binding domains that facilitate their crosslinking and binding functions (Figure 1). More specifically, the spectraplakins MACF1 and BPAG1 are comprised of an actin-binding domain, a plakin domain, an EF-hand calcium-binding domain, as well as a spectrin-repeat rod and growth-arrest specific 2-related microtubule-binding domain, present only in spectraplakins (Figure 1). In contrast, the structural organization of envoplakin, periplakin, plectin, and desmoplakin are less complex than that of spectraplakins. These cytoskeletal binding proteins primarily consist of a plakin domain, a coiled-coil rod, and plectin repeat domains. Of note, plectin is the only non-spectraplakin with an actin-binding domain, while epiplakin is comprised only of plakin-repeat domains. The structural organization and functional domains of mammalian plakins have been reviewed further elsewhere [8,9]. 

The multifarious structural organization of mammalian plakins is paralleled by their diverse biological roles. MACF1 and BPAG1 have both been described to play roles in cell migration and microtubule organization [10,11,12,13,14], while desmoplakin, envoplakin, and periplakin have been shown to play a prominent role in the development of the cornified envelop, a layer under the plasma membrane of keratinocytes in the outermost layers of the epidermis [4,15]. To date, desmoplakin is the best characterized plakin in terms of its numerous biological roles, which include involvement in myocardium morphogenesis [16,17], epidermis development [18], keratinocyte differentiation [19,20], and wound healing, in which MACF1 has also been shown to play a role [21,22]. 

**Figure 2:**
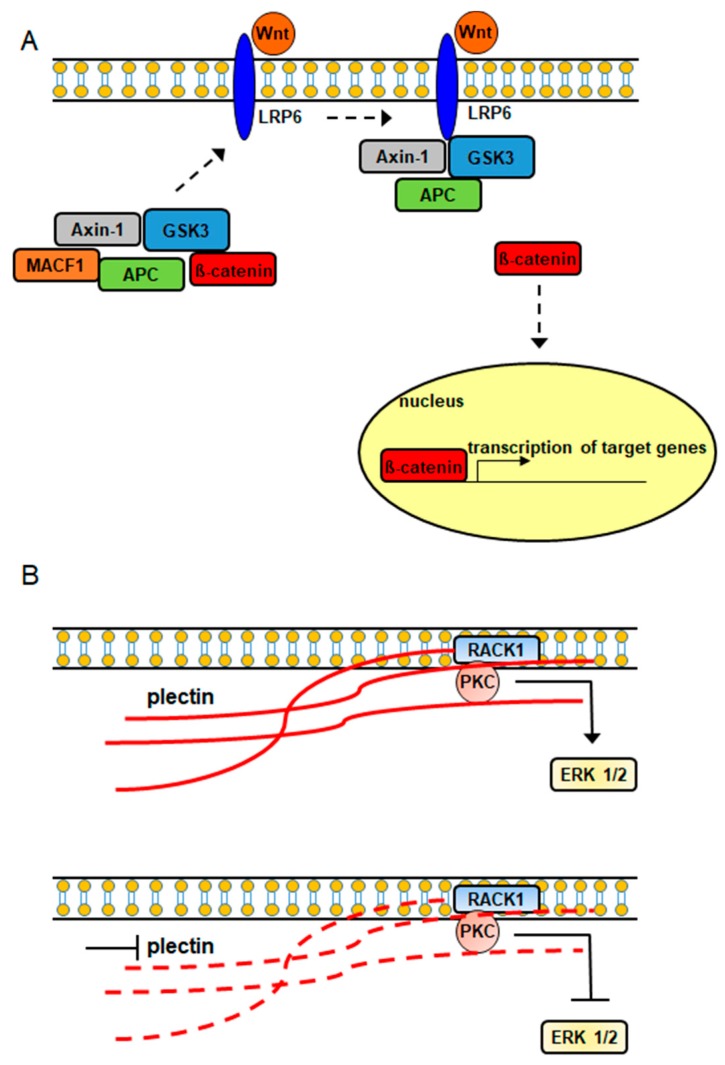
Plakin proteins participate in intracellular signaling cascades. (**A**) MACF1–Wnt signaling (dashed arrow: WNT signaling mediators interacting with WNT receptor), (**B**) Plectin–PKC–ERK signaling (solid red line: intact plectin; dashed red line: inhibited down-regulated plectin).

It has also been demonstrated that plakins are involved in intracellular signaling cascades (Figure 2). In a study conducted by Chen et al., (2006) it was shown that MACF1 played a role in the Wnt signaling axis via its involvement in the translocation of the Wnt signaling mediator, Axin1, to the plasma membrane, where it interacts with the Wnt signaling receptor LRP6 (Figure 2), while Hamill et al., demonstrated that the signaling interaction of Rac1 with β4 integrin decreased in keratinocytes lacking BPAG1 [23,24]. Furthermore, plectin was shown to complex with the receptor for activated C kinase 1 (RACK1) and with protein kinase C in yeast two-hybrid and immunoprecipitation experiments [25]. Additionally, the downregulation of plectin using RNA interference was accompanied by decreased expression of the pleiotropic signaling protein extracellular signal-regulated kinases ½ [26]. Because RACK1 is an adapter protein for protein kinase C, which is an upstream signaling regulator of extracellular signal-regulated kinases ½, it is possible that plectin’s signaling association with extracellular signal-regulated kinases ½ is mediated via its direct binding with RACK1 and protein kinase C (Figure 2). 

Given the compositional diversity of plakin proteins and the abundance of biological and mechanistic roles they have in several cellular and signaling processes, along with the evidence that shows that knocking out genes of several plakin family members is lethal in early stages of mice development, it is suggested that genetic and functional aberrations of these macromolecules contribute to diseases and pathological conditions (Table 1) [25,27,28].

## 2. Plakins and Disease

### 2.1. Skin, Heart, and Neurological Diseases

Bullous pemphigoid was first described by Lever in 1953 and is an acute and chronic autoimmune skin disease that primarily affects elderly people and involves the formation of blisters (bullae) [59]. The clinical manifestation of bullous pemphigoid is the result of antibodies targeting BPAG1 or BPAG2 (collagen alpha-1 XVII) between the epidermis and dermis skin layers. Bullous pemphigoid has an incidence rate that ranges from 2.5 to 42.8 cases per one million persons a year, with an equally varied mortality rate that has been documented to range from 6% to 41% [36]. Like BPAG1, desmoplakin has also been shown and described to be involved in several pemphigoid autoimmune skin disorders (paraneoplastic pemphigus, pemphigus foliaceus, erythema multiforme, and mucosal-dominant pemphigus vulgaris) including bullous pemphigoid, whose pathology is associated with antibodies binding to desmoplakin, and which presents with blistering [37,38,39]. Additionally, gene mutations in desmoplakin have been documented to underlie palmoplantar keratoderma [60], a thickening of the skin on the palms of the hands and soles of the feet. Desmoplakin mutations have also been identified as a cause of dilated cardiomyopathy and arrhythmogenic right ventricular cardiomyopathy [43,44,45,46,47]. The mortality rate for arrhythmogenic right ventricular cardiomyopathy has been stated to be between 2–4% per year, and the survival rate for dilated cardiomyopathy is reported as <50% at 10 years postdiagnosis [61]. Although little is known regarding the role of periplakin and envoplakin in human diseases and skin disorders, these plakins have been implicated in paraneoplastic pemphigus as a consequence of an autoimmune response to these proteins, like that observed for BPAG1 and desmoplakin in the related diseases [53,54]. A cumulative autoimmune response to periplakin, envoplakin, BPAG1, and desmoplakin has been described in persons affected by paraneoplastic pemphigus that results in a dismal prognosis with a 75–90% mortality rate and a mean survival < 1 year [62]. Consistent with other plakin family members, genetic abnormalities in plectin have also been described to contribute to the skin disorder epidermolysis bullosa simplex [57].

Plakin proteins, specifically the spectraplakins BPAG1 and MACF1, have also been associated with neurological-related disorders. BPAG1 was described as an immunogenic target in multiple sclerosis patients, an autoimmune disease of the nervous system that effects the myelin sheath in the peripheral nervous system. In this regard, Laffitte et al. (2005) provided evidence that the cerebral spinal fluid of patients with multiple sclerosis contained BPAG1 antibodies, as determined by their reactivity to recombinant BPAG1 proteins [40]. The involvement of BPAG1 in neurological diseases is further evidenced by in vivo mice studies that established that BPAG1 mutations resulted in the neurodegenerative disease dystonia musculorum, while its overexpression reversed the diseased phenotype [41,42]. The duplication of a region on chromosome 1p34.3 where MACF1 is located was identified as the only chromosomal abnormality detected in a patient suffering from a neuromuscular condition [29], while genetic variations of MACF1 have been correlated with increased risk of Parkinson’s disease [30]. 

### 2.2. Cancer and Plakins

Plakins have also been found to play various roles in cancer. The clinical association of plakins in cancer can be observed in part in paraneoplastic pemphigus and bullous pemphigoid patients, who have also been diagnosed with hematopoietic and lymphoid cancers [63,64,65,66]. Although the etiological pathology and causal relationship of these diseases is still unclear, the role of several plakin molecules in cancer has been characterized, most notably the involvement of desmoplakins in neoplasms. The expression analysis of desmoplakin in several cancers showed decreased levels or the absence of this plakin in oral squamous cell carcinomas and carcinomas of the lung [48,49]. It was also shown that desmoplakin expression was reduced in metastatic oropharyngeal cancers, and that desmoplakin (II) expression, a product of alternative splicing, in these cancers was associated with a poor clinical outcome [50]. These data provided evidence that desmoplakin may serve as a diagnostic and prognostic cancer biomarker and perform tumor suppressor functions. Further evidence for the role of desmoplakin as a tumor suppressor was provided in a study by Wan et al. (2007) who demonstrated that the downregulation of desmoplakin increased keratinocyte proliferation and induced the expression of the mitogenic prosurvival signaling mediators ERK1/2 and Akt [51]. Contrary to the observation that antagonizing desmoplakin function promotes keratinocyte proliferation, the overexpression of desmoplakin was described to inhibit lung cancer cell proliferation and was accompanied by the downregulation of the Wnt signaling mediator Axin2 [52]. Taken together, these experimental studies provide additional context to the role of desmoplakin as a tumor suppressor. Like desmoplakin, periplakin’s loss of expression has also been affiliated with the development and progression of cancer, specifically, bladder, colon, and esophageal squamous cell carcinoma cancers [55,67,68]. Similar to desmoplakin and its tumor suppressor characteristics, the overexpression of periplakin suppressed the proliferation of colon cancer cells and reduced the expression of pro-cell cycle regulators [55]. From a mechanistic standpoint, periplakin hypermethylation, which was observed to be higher in esophageal squamous cell carcinomas as compared to the normal tissue by bisulfite pyrosequencing, has emerged as an underlying epigenetic contributor to the reduced periplakin expression that compromises its tumor suppressor functions [56]. 

The characterization of MACF1 function in cancer has been primarily limited to high-throughput genomics and proteomics approaches. One of the first studies linking MACF1 to cancer was performed using alternative splicing microarray profiling that revealed transcript alterations of MACF1 in non-small cell lung cancer [31]. A subsequent investigation using proteomics techniques identified MACF1 as a potential oncofetal biomarker that may be used for the diagnosis of colorectal cancer [32], while, most recently, whole-exome sequencing described MACF1 mutations in patients with endometrial cancer and renal cell carcinomas [33,34]. Few investigations to date have examined MACF1 function in cancer biology. To this end, our laboratory has provided evidence that MACF1 is expressed in glioblastomas but not in normal brain tissue and lower-grade brain tumors [35]. Furthermore, our work revealed that downregulating MACF1 expression reduced glioblastoma cell proliferation and migration, which was also accompanied by reductions in Axin and β-catenin expression, which are mediators of Wnt signaling [35]. These data provide evidence that MACF1 is a potential oncoprotein and diagnostic cancer marker, at least in brain tumors. In contrast to other plakin proteins, little is known regarding BPAG1 and its role in cancer. However, in a study in melanoma, BPAG1 auto-antibodies were observed in patients with early and advanced stages of this disease as compared to normal individuals [69]. Lastly, plectin was also described as a potential biomarker in pancreatic cancer, where expression of this plakin was observed in primary and metastatic pancreatic ductal adenocarcinomas [58].

## 3. Microtubule-Targeted Drug Therapy

The clinical utility of drugs targeting cytoskeletal proteins for the treatment of diseases, specifically cancer, has primarily been demonstrated for microtubule-targeting agents. Comprised of heterodimers of α- and β-tubulin, microtubules provide structure and stability in cells, essential for mitotic spindle formation during cell division and vesicular and organelle transport. The kinetic instability of microtubules, as a consequence of polymerization and depolymerization during transitional cellular processes, have made them practical pharmacological targets in diseases like cancer, where high cellular proliferation rates and migratory invasive processes that depend on microtubule function underlie disease pathology and progression. Typically, three classes of microtubule inhibitory agents are used for the clinical treatment of cancers: vinca alkaloids, colchicines, and taxanes [70,71,72]. Vinca alkaloids, which consist of vincristine and vinblastine, along with colchicines employ their antimicrotubule effects by preventing tubulin polymerization as a consequence of binding at the ends of microtubules and at the α–β tubulin heterodimer interface, respectively. Taxanes, consisting of the microtubule-targeting agents paclitaxel and docetaxel, provoke their antimicrotubule effects by inhibiting microtubule disassembly as a result of interacting with β tubulin subunits. It should also be mentioned that a number of contemporary microtubule-targeting agents have been evaluated for their therapeutic efficacy. These include, but are not limited to, discodermolide, eleutherobin, estramustine, and epothilones [73,74]. Clinical assessments on the effectiveness of microtubule-targeting agents used to treat several cancers have established that the use of these drugs as first-line single agent therapies or as part of a combinatorial treatment regimen is cancer-dependent. Specifically, breast cancers were shown to have a better overall response rate when treated with taxanes in combination with other chemotherapeutic agents (anthracycline or carboplatin) than when treated with taxanes alone [75,76]. Urothelial tumors have proven to be one of the few cancers that demonstrated a relatively positive tumor response to a single-agent treatment with the taxane paclitaxel, as indicated by a 42% tumor response rate [77]. Like taxanes, the utility of vinca alkaloids, as single agents or as part of a synergistic therapeutic drug approach, is also cancer-dependent. Procarbazine, lomustine, and vincristine compose a multidrug combination used for the clinical treatment of recurrent high-grade gliomas, that demonstrated that patients treated with this regimen had a median survival of six months [78]. The prognosis of small-cell lung cancer patients treated with a combination of a cyclophosphamide, epirubicin, and vincristine also had poor outcomes with a two- and five-year survival rates of 8% and 3%, respectively [79]. In contrast, a vincristine multidrug combination used to treat non-Hodgkin lymphoma patients was described to be curative [80], while vinca alkaloids as a single first-line therapy for the treatment of mesothelioma has been suboptimal [81]. Despite the differential impacts of various treatment protocols that use microtubule-targeting agents, a major caveat to the clinical effectiveness of these drugs has been toxicity, with neurological and hematological toxicities being the most prevalent [82,83]. 

Experimental investigations by us and others have also provided insight on the effects of microtubule-targeting agents on plakin proteins, specifically MACF1, which interacts with microtubules via its microtubule-binding domain. In a study by Sun et al., it was determined that the GAR–GSR microtubule-binding domain of MACF1 was critical for stabilizing microtubules after exposure to the microtubule-interacting agent nocodazole in COS7 monkey kidney cells, while MACF1 expression appeared to be redistributed in response to epothilone B [84,85]. These data suggest that, although MACF1 is not a direct target of microtubule-stabilizing or -inhibiting agents, it is indirectly affected by and differentially responsive to these drugs.

## 4. Therapeutic Targeting of Plakins

The current standard of therapy for treating diseases related to genetic malformations or abnormal autoimmunological responses to plakin proteins are corticosteroids and antibiotics, neither of which are specific for these cytoskeletal cross-linkers. At present, there are no known experimental or clinical pharmacological agents targeting plakin protein family members, despite their involvement in heart disease and cancer, the top-two leading causes of death in the United States [86]. Given the absence of plakin-specific drugs, a bioinformatics approach was used to identify drugs that interact with these cytoskeletal linkers. The Research Collaboratory for Structural Bioinformatics (RCSB) protein data bank was used to identify 329 ligands predicted to interact with plakin family members. Of the 329 identified, 106 were ligands of MACF1, 88 of BPAG1, 91 of plectin, 11 of desmoplakin, 18 of envoplakin, 9 of periplakin, and 6 of epiplakin [87]. 

**Figure 3:**
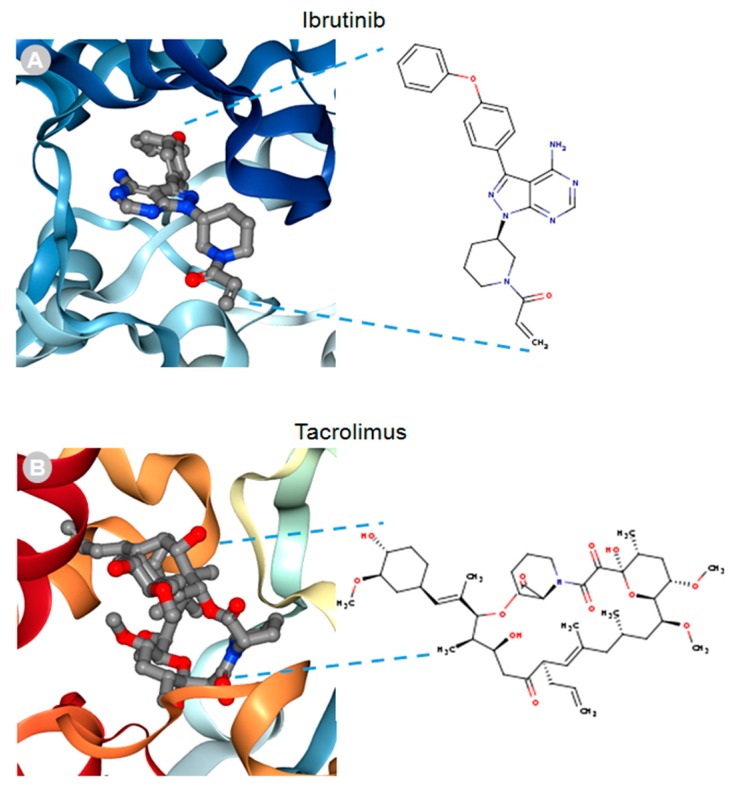
Ibrutinib (**A**) and tacrolimus (**B**) drug ligands interacting with 3D ribbon structures of calcium-dependent protein kinase 1 and calcineurin A and B proteins, respectively, indexed in the Research Collaboratory for Structural Bioinformatics protein data bank (RSCB PDB). Images produced in the RSCB PDB [87]. Figure 3 and Figure 4 do not need copyright permission only to be cited as has been done.

**Figure 4:**
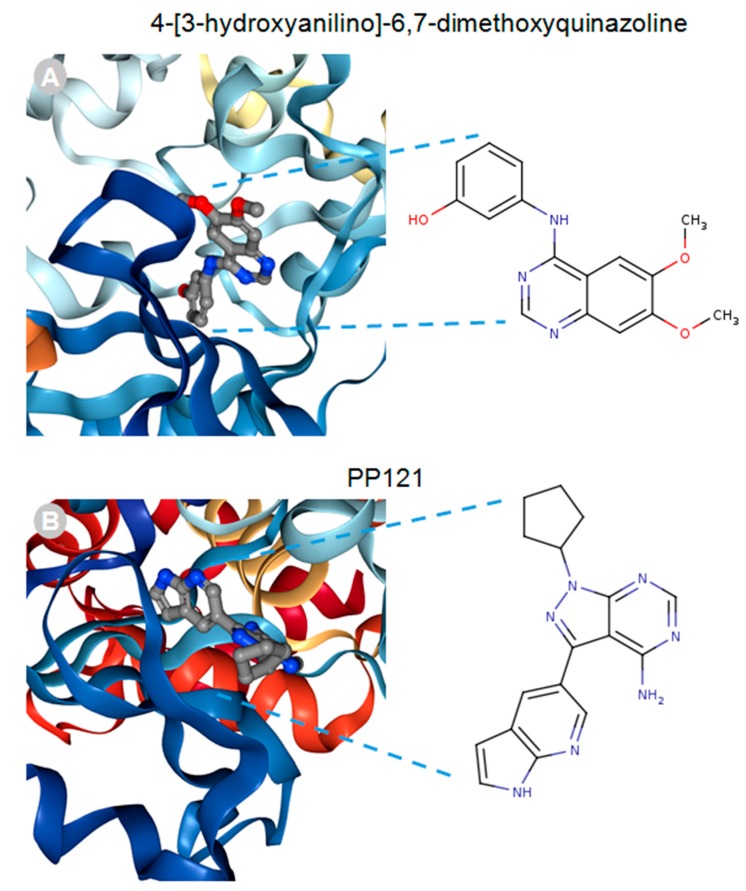
4-[3-hydroxyanilino]-6,7-dimethoxyquinazoline (**A**) and PP121 (**B**) drug ligands interacting with the calcium-dependent protein kinase 1, 3D ribbon structure indexed in the RSCB PDB. Images produced in the RSCB PDB [87].

MACF1 and BPAG1, the only plakin proteins with microtubule-binding domains, were predicted to interact with 60% of the identified plakin ligands and to share several common ligands [87]. One of these was the small molecule tyrosine kinase inhibitor, ibrutinib (Figure 3), whose mechanism of action directly impairs Bruton’s tyrosine kinase (BTK) function through competitive inhibition and negatively regulates its capacity as a multifaceted intracellular signaling molecule [88]. Ibrutinib is used clinically for the treatment of lymphoma and leukemia and has also been suggested to improve the progression-free survival and overall survival of chronic lymphocytic leukaemia [89,90,91,92]. Despite the enthusiasm of this drug agent for the treatment of these malignancies, it has also been shown to cause several associated toxicities that include diarrhea, bleeding, neutropenia, and anemia [93]. Like ibrutinib, the macrolide antibiotic immunosuppressive agent tacrolimus was also identified as a ligand of MACF1 and BPAG1 (Figure 3). Tacrolimus, which inhibits the phosphatase activity of calcineurin and the gene transcription of several cytokines (IL-3, IL-4, IL-5, GM-CSF, and TNF), is used as a topical agent for the clinically treatment of atopic dermatitis and as an immunosuppressive drug for postoperative organ transplant to minimize organ rejection [94,95,96,97,98,99,100,101]. Most recently, a meta-analysis suggested that tacrolimus enhanced the clinical symptoms of the autoimmune neuromuscular disease myasthenia gravis, with 23% of patients displaying adverse effects [102]. Also among the shared ligands of MACF1 and BPAG1 is 4-3-hydroxyanilino-6,7dimethoxyquinazoline (Figure 4), which belongs to the class of quinazoline molecules and is known to have several biomedical applications as an antibacterial, antifungal, anti-inflammatory, antimalaria, antiviral, anticancer, and antituberculosis agent. 

Notable quinazolines are the anticancer agents iressa and erlotinib, whose principal mechanistic function is the inhibition of the epidermal growth factor receptor, as well as 2-styrylquinazolin-4(3H) quinazolines that exhibit anticancer effects by inhibiting microtubule polymerization [103,104,105,106]. PP121, a dual tyrosine kinase and phosphoinositide kinase inhibitor that interferes with PI3K and mTOR function, was determined to be a MACF1 ligand (Figure 4) [107]. Although no clinical studies have been performed regarding the efficacy of PP121, several experimental investigations have provided evidence of its potential clinical applications. In studies by Peng et al. (2015) and Che et al. (2014), PP121 was shown to have antitumorigenic effects on esophageal cancer and anaplastic thyroid carcinoma, as a consequence of perturbing PI3K and mTOR kinase activities [108,109]. Even more significant, was the observation that PP121 was not toxic to the normal human esophageal cells, suggesting that PP121 specifically targets cancer cells and may have minimal toxic side effects [108]. 

**Figure 5:**
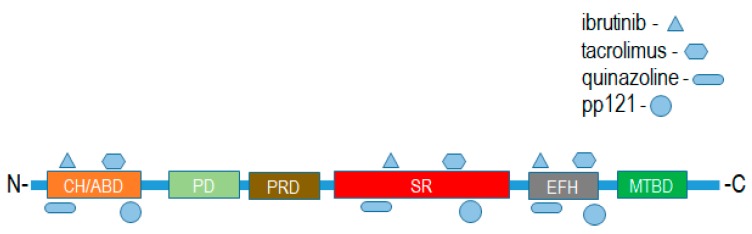
Putative binding sites of drug ligands with domains of spectraplakin proteins. CH/ABD:Calponin homology actin-binding domain; PD: plakin domain; PRD: plakin repeat domain; SR: spectrin repeat; EFH: EF-hand calcium-binding motif region; MTBD: microtubule-binding domain.

Putative binding interactions between spectraplakin proteins and identified ligands that bind them were determined using ligand–target sequence analysis and indexed ligand–macromolecule associations within the RSCB protein data bank (Figure 5). Binding interaction analyses revealed that the identified ligands of MACF1 and BPAG1, discussed above, associated with varied domains that comprise these cytoskeletal linkers (Figure 5). Specifically, spectraplakin-interacting ligands were determined to associate with the calponin homology region at the amino-terminus, and spectrin repeats and EF-hand calcium-binding regions at the carboxyl terminus of MACF1 and BPAG1 (Figure 5).

**Figure 6:**
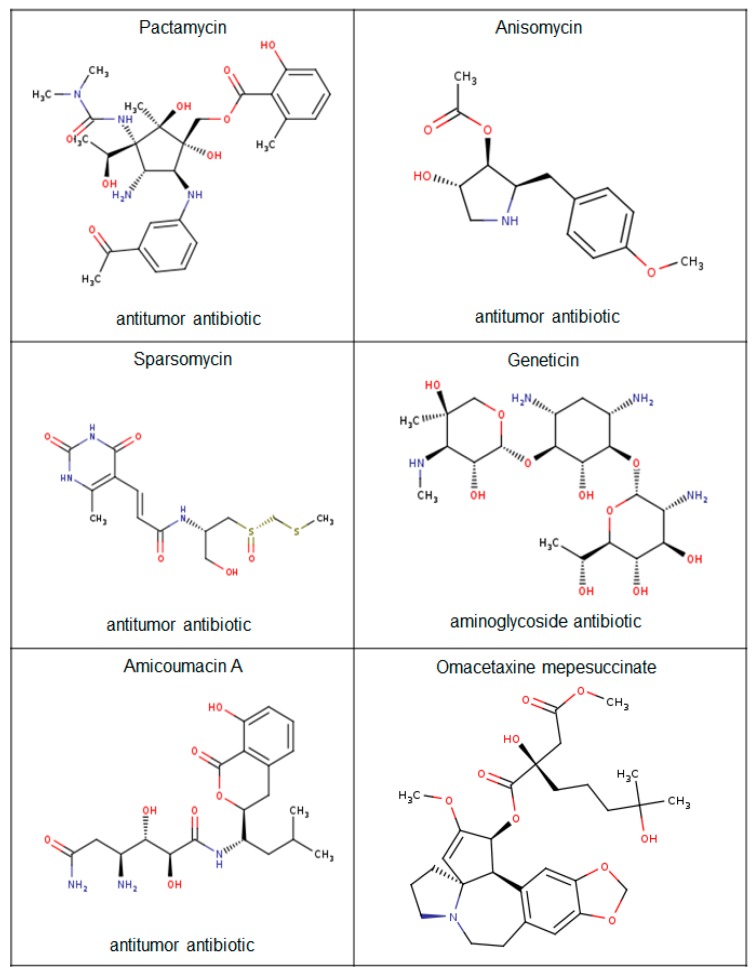
Plectin anticancer antibiotic ligands.

Further analysis of plakin proteins revealed that plectin was a substrate for the antibiotics sparsomycin, pactamycin, and amicoumacin A and for the cephalotaxine omacetaxine mepesuccinate (Figure 6). These compounds all share a similar mechanistic inhibitory function that typically involves prohibiting translation by binding to transfer-RNA docking sites within the large ribosomal subunit and have all been described to have antitumorigenic properties.

Amicoumacin A was recently shown to have cytotoxic effects on breast cancer and lung cancer cells, while pactamycin had a cytostatic antitumorigenic effect on head and neck squamous cell carcinomas as a result of the induction of several cell cycle arrest regulatory proteins [110,111]. Sparsomycin, another cytostatic agent, also inhibited the growth of murine leukemia and renal cell carcinomas [112,113]. The most clinically relevant plectin ligand is omacetaxine mepesuccinate that is used to treat patients with chronic myeloid leukemia that are resistant to tyrosine kinase inhibitors [114,115,116]. In support of this, Cortes et al. (2015) provided clinical data that showed that omacetaxine mepesuccinate increased the median overall survival of chronic myeloid leukemia patients [117].

Albeit unlikely that the plakin drug ligands described above would be clinically efficacious, as a consequence of lacking target specificity to plakin proteins, derivative compounds of the identified plakin drug ligands discussed here could prove to have a beneficial therapeutic value. This could particularly be advantageous in treating a disease like cancer, for which evidence exists that plakin proteins such as MACF1 and plectin may act as oncoproteins. It is conceivable that analogues of the plakin ligands identified here could specifically target and inhibit MACF1 and plectin function, consequently impairing their direct binding interactions with Wnt and ERK1/2 signaling mediators, respectively, which regulate protumorigenic signaling pathways.

## 5. Conclusions

**Figure 7:**
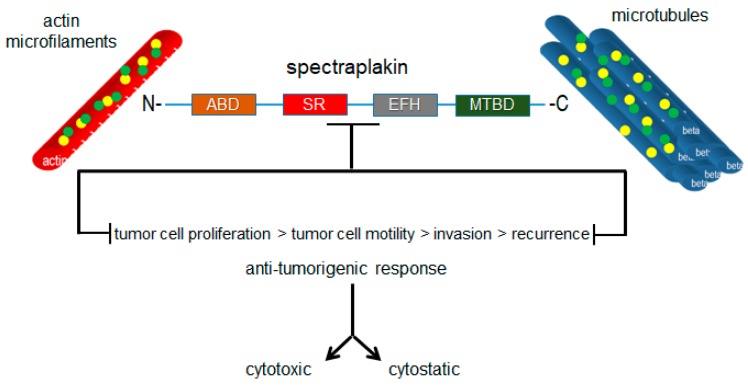
Targeted inhibition of plakin proteins impairs the crosslinking functions necessary for tumorigenic cell behaviors and responses.

Plakins are structurally and functionally diverse proteins, as reflected across the range of diseases in which they have been established to play roles. However, they have not been considered as cytoskeletal drug targets with therapeutic value for the development of drugs targeting these proteins, particularly those with the potential to be oncoproteins such as MACF1. Microtubules and microtubule-targeting agents have been given this type of consideration. However, as widely acknowledged, the clinical effectiveness of microtubule-targeting agents is minimized because of the associated toxicities and is compounded by tubulin mutations and isotypes that compromise the ligand–substrate interaction, and thus their efficacy. The efforts to improve the usefulness of microtubule poisons have in part focused on the discovery of compounds in novel natural products that circumvent binding to drug efflux transporters and on the synthesis of chemical modifications in existing microtubule agents to improve their tubulin-binding affinity. Albeit pragmatic, this does little with respect to improving the toxicity profile and target-specific effects of microtubule-targeting agents in diseased tissues. Plakins, which possess several genetic alteration frequencies and aberrant expression profiles in a number of diseases, represent a potentially new class of therapeutic targets that could expand targeted-therapy approaches by exploiting the inter- and intradisease diversity of the pathologies in which these proteins participate. It is also conceivable that drugs developed to target plakin family members will have collateral impacts on numerous cell behaviors that contribute to the development and progression of various diseases, such as cancer, because of their convergent crosslinking functions in these processes (Figure 7). Furthermore, the observation that plectin is a ligand for several antitumor antibiotics, also encourages investing in the development of plakin-targeted drugs and broadens the spectrum of cytoskeletal proteins that can be therapeutically targeted. 

## Figures and Tables

**Table 1:** Diseases and disorders associated with plakin proteins.

Plakin Family Member	Associated Diseases and Disorders	Refs.
MACF1	Neuromuscular disease, Parkinson’s disease, cancer	[29,30,31,32,33,34,35]
BPAG1	paraneoplastic pemphigus, pemphigus foliaceus, erythema multiforme, mucosal-dominant pemphigus vulgaris, multiple sclerosis, dystonia musculorum	[36,37,38,39,40,41,42]
Desmoplakin	paraneoplastic pemphigus, pemphigus foliaceus, erythema multiforme, mucosal-dominant pemphigus vulgaris, cardiomyopathy, cancer	[37,38,39,43,44,45,46,47,48,49,50,51,52]
Envoplakin	paraneoplastic pemphigus	[53,54]
Periplakin	paraneoplastic pemphigus, cancer	[53,54,55,56]
Plectin	epidermolysis bullosa simplex, cancer	[57,58]
